# Progesterone receptor is constitutively expressed in induced Pluripotent Stem Cells (iPSCs)

**DOI:** 10.1007/s12015-024-10776-6

**Published:** 2024-08-22

**Authors:** Michele Manganelli, Elena Laura Mazzoldi, Rosalba Monica Ferraro, Marinella Pinelli, Marta Parigi, Seyed Ali Mir Aghel, Mattia Bugatti, Ginetta Collo, Gabriele Stocco, William Vermi, Stefania Masneri, Camillo Almici, Luigi Mori, Silvia Giliani

**Affiliations:** 1 Angelo Nocivelli Institute for Molecular Medicine, Department of Molecular and Translational Medicine, University of Brescia, ASST Spedali Civili, 25123 Brescia, Italy; 2 Department of Molecular and Translational Medicine, University of Brescia, ASST Spedali Civili, 25123 Brescia, Italy; 3 Division of Farmacology, Department of Molecular and Translational Medicine, University of Brescia, 25123 Brescia, Italy; 4 Clinical Department of Medical, Surgical and Health Sciences, University of Trieste, 34127 Trieste, Italy; 5 Institute for Maternal and Child Health, IRCCS Burlo Garofolo, 34127 Trieste, Italy; 6 Division of Immunohematology and Transfusion Medicine, ASST Spedali Civili Di Brescia, 25123 Brescia, Italy; 7 Department of Clinical and Experimental Sciences (DSCS), University of Brescia, 25123 Brescia, Italy; 8 SSVD Laboratory of Medical Genetics, ASST Spedali Civili, 25123 Brescia, Italy

**Keywords:** CD34, Fibroblasts, Induced pluripotent stem cells (iPSCs), Progesterone receptor, Estrogen receptor, Differentiation

## Abstract

Induced Pluripotent Stem Cells (iPSCs) are nowadays a common starting point for wide-ranging applications including 3D disease modeling (i.e. organoids) and in future regenerative medicine. Physiological processes like homeostasis, cell differentiation, development and reproduction are tightly regulated by hormones through binding to their transmembrane or nuclear receptors of target cells. Considering their pleiotropic effect, take into account also their expression in an iPSCs-based disease modeling would better recapitulate the molecular events leading to 3D organoid development and disease study. Here we reported the expression pattern of estrogen receptor (ERα) and progesterone receptor (PR) in four different iPSCs, obtained from CD34 + progenitor cells and skin fibroblasts with four different methods. Expression of ERα and PR mRNA were significantly downregulated in iPSCs as well as fibroblasts compared to MCF7 positive control. Immunofluorescence (IF) staining detected only the expression of PR protein in all the different iPSCs cell lines, while ERα was not detectable. By flow cytometry analysis we observed that the ~ 65% of the total population of iPSCs cells expressed only PR, with 100% fold increase compared to HSPCs and fibroblasts, while ERα was not expressed. Our results collectively demonstrated for the first time that the reprogramming of somatic cells into iPSCs leads to the expression of PR receptor.

**Graphical Abstract:**

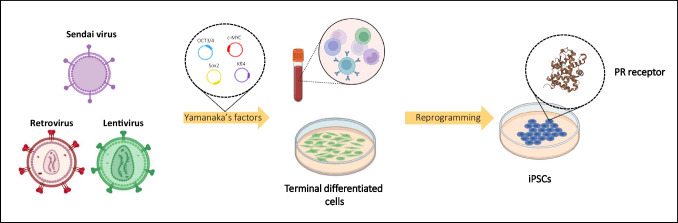

**Supplementary Information:**

The online version contains supplementary material available at 10.1007/s12015-024-10776-6.

## Introduction

Induced Pluripotent Stem Cells (iPSCs) can differentiate in vitro and in vivo into various cell types, enabling the development of an unlimited source of almost any type of human cells.

Since 2006, when Yamanaka and colleagues first generated iPSCs, reprogramming technologies have significantly progressed [1]. In addition to lenti- or retroviral mediated integrative transgene delivery strategies, many different methods to introduce exogenous reprogramming factors (*Oct4, Sox2, Klf4, c-Myc*) into the cells have been established to improve reprogramming efficiency and to generate transgene-free iPSCs for potential iPSCs-based cell technology. These reprogramming methods included the delivery of transgenes by using non-integrating viral approaches as the Sendai virus, or non-viral methods such as episomal vectors, mini-circle DNA vectors, piggy-Bac transposons, synthetic mRNAs, or recombinant cell-penetrating proteins [2, 3].

Moreover, the maintenance of genomic integrity of iPSCs would promote the development of a useful platform and a powerful tool for a wide range of biomedical applications including, but not limited to, drug development, disease modeling, tissue engineering and regenerative medicine [4, 5]. Among the different fields of iPSCs applications, one of the last frontiers is the development of organoids, 3D structures that spontaneously self-organize into adequately differentiated cell types, aimed to recapitulate the functions of the target organ. Indeed, the application of iPSCs in intestinal [6], liver [7], brain [8], kidney [9] and breast organoids [10] has been extensively reported.

Currently, blood cells and skin fibroblasts are the most used cell types for reprogramming because they are easy to obtain (skin biopsy, blood sample), conditions for their culture are well established, and reprogramming methods for iPSCs are successfully standardized. Accordingly, when non-mobilized peripheral blood (PB) samples are used, many protocols include cultivation steps with various combinations of cytokines to preserve the viability of CD34 + hematopoietic stem/ progenitors cells (HSPCs) cells and thus the reprogramming efficiency [11–15].

Steroid hormones, as estrogen and progesterone, regulate a wide range of physiological processes including cell differentiation and development, cellular homeostasis and reproduction [16, 17]. Estrogens are mainly produced from androgens precursors in ovarian granulosa cells and placenta in females, testis in males and non-glandular tissue (fat and bone) in both sexes [18]. Progesterone is produced by ovarian granulosa cells, adrenal glands, corpus luteum during the menstrual cycle and placenta in females and by adrenal glands in both males and females [19]. They exert their function by binding to intracellular receptors (ERα/-β and PR), members of the nuclear receptor superfamily of transcriptional co-activators [20]. Estrogen and progesterone play different functions during embryonic development [21]. After birth, they also control the maturation of immune cells, exerting significant effects on the bone marrow (BM), on hematopoietic stem (HSCs) and progenitor cells in particular, for the development and maturation of the hematopoietic lineages [22–27]. Considering their pleiotropic effects, it is important to understand their role also during cell differentiation, in particular for those in vitro disease modeling which include steroid hormone cellular response such as reproductive organs (i.e. ovaries, breast).

Here we reported the expression patterns of estrogen receptor-α (ERα) and progesterone receptor (PR) in four iPSCs cell lines obtained with four different reprogramming methods to better define the possible role of steroid-hormone receptors in iPSCs-based cell differentiation modeling.

## Materials and Methods

### Cell Culture

Human neonatal foreskin fibroblasts (BJ strain, purchased from ATCC), MCF7 (ERα + /ERβ-/PR +) and MDA-MB-231 (ERα-/ERβ + /PR-) were grown in DMEM (Euroclone S.p.a., Pero, Italy) supplemented with 10% fetal bovine serum (FBS, Euroclone S.p.a.), 1% L-Glutamine, and 1% Penicillin/Streptomycin (Euroclone S.p.a.). In this study we used four different iPSCs cell lines (Table 1), one of them purchased from ThermoFisher Scientific, Inc. (cat. n. A18945, ThermoFisher Scientific, Inc., Waltham, MA, USA) obtained from cord-blood CD34 + progenitors’ cells, and three of them from skin fibroblasts.

**Table 1:** List of iPSCs cell lines used in this study

iPSCs cell line	Parental cell	Reprogramming method	Ref
Episomal (cat. n. A18945)	cord blood-derived CD34 + progenitors	Episomal Vector	[28]
BJ	human foreskin fibroblasts	CytoTune-iPS 2.0 Sendai Reprogramming Kit	[29, 30]
253-G1	human fibroblasts	Retroviral trasduction	[31]
F3	human fibroblasts	Lentiviral trasduction	[32, 33]

Each iPSCs cell line was generated performing four different independent reprogramming methods, as previously reported [28–33]. iPSCs were fed daily with NutriStem® hPSC XF Medium (Sartorius AG, Göttingen, Germany) with the addition of 10 ng/ml of bFGF (Basic fibroblast growth factor; Miltenyi Biotec GmbH., Bergisch Gladbach, Germany), manually picked every 4–5 days on new Matrigel-coated well plate (Corning Inc., Corning, NY, USA) and cultured at 37° C in 5% CO_2_.

### RNA Extraction and qPCR

Total RNA was extracted using NucleoSpin® RNA II kit (Macherey–Nagel, Düren, Germany), treated for TURBO™-DNase digestion (Invitrogen; ThermoFisher Scientific, Inc.) and quantified by a spectrophotometer (Tecan Group Ltd., Männedorf, Switzerland). One µg of total RNA was retro-transcribed by ImPromII™ Reverse Transcription System (Promega Corporation, Madison, Wisconsin, USA), following the manufacturer’s protocol. qPCR gene expression analysis was performed using SYBR Green (Bio-Rad Laboratories, Inc., Hercules, CA, USA). Primer pairs (IDT, Inc., Coralville, IA, USA) used in this study are listed in Table2.

**Table 2:** List of primers used in this study

Primer name	Primer Sequence (5′3’)
CK5_F	CATGGACAACAACCGCAACC
CK5_R	ACTGCTACCTCCGGCAAGAC
CK7_F	AGGAGAGCGAGCAGATCAAG
CK7_R	CAGAGATATTCACGGCTCCC
CK18_F	TGGCAATCTGGGCTTGTAGG
CK18_R	AGAACGACATCCATGGGCTC
GATA3_F	TCATTAAGCCCAAGCGAAGG
GATA3_R	GTCCCCATTGGCATTCCTC
TP63_F	CTTGCCCAGGAAGAGACAGG
TP63_R	CATAAGTCTCACGGCCCCTC
ERα_F	CCACCAACCAGTGCACCATT
ERα_R	GGTCTTTTCGTATCCCACCTTTC
ERβ_F	AGAGTCCCTGGTGTGAAGCAA
ERβ_R	GACAGCGCAGAAGTGAGCATC
PR_F	CGCGCTCTACCCTGCACTC
PR_R	TGAATCCGGCCTCAGGTAGTT

The thermocycler conditions were 98 °C for 30 s, 39 cycles of 95 °C for 5 s and 60 °C for 30 s, followed by 65 °C for 5 s. Assays were performed on CFX96 C1000 Touch™ Real-Time PCR Detection System and analyzed with CFX manager software v.3.1 (Bio-Rad Laboratories, Inc.). Gene expression was quantified as fold change, wherein the ΔCt values were calculated by subtracting the average Ct value of the target gene from the average Ct value of β-actin (Hs.PT.56a.19461448.g; IDT, Inc.) used as reference gene. Data were generated from at least three independent experiments.

### Immunofluorescence (IF)

iPSCs were fixed and permeabilized using Fix&Perm-Reagent kit (Nordic-MUbio, Susteren, The Netherlands) according to the manufacturer’s instructions. Then, blocking solution iBind™ Buffer (Invitrogen; ThermoFisher Scientific, Inc.) was applied for 30 min. Primary antibodies for ER (clone SP1-rabbit, ready to use, Ventana, Roche, Basel, Switzerland), PR (clone 1E2-rabbit, ready to use, Ventana, Roche), CD44 (1:50, clone DF1485-mouse, Dako, Glostrup, Denmark), and Alexa Fluor-488-phalloidin (1:500; Merck KGaA, Darmstadt, Germany), and secondary antibodies (1:250; goat anti-mouse and anti-rabbit IgG (H + L) Alexa Fluor-568; ThermoFisher Scientific, Inc.) were added and incubated for 2 h, at room temperature (RT). Cellular nuclei were counterstained with DAPI for 5 min. Cells were observed with an inverted fluorescence microscope (Olympus IX70, Olympus Optical Co., GmbH, Hamburg, Germany), and images were analyzed with the Image-Pro Plus software v7.0 (Media Cybernetics, Inc., Rockville, MD, USA).

### Flow Cytometry Analysis

Five mobilized-PB samples with G-CSF (Granulocyte colony-stimulating factor) for transplant at ASST Spedali Civili of Brescia were collected as control group. Informed consent was obtained from all the subjects enrolled in this study. The PB was collected in EDTA tubes for CD34 + cells- evaluation. Briefly, 1 ml of blood was treated with BD Pharm Lyse™ Lysing Buffer (Becton, Dickinson and Company, Franklin Lakes, NJ, USA), according to the manufacturer’s instructions. After red blood cells lysis, cells were centrifuged at 1600 rpm for 4 min and labeled for flow cytometry analysis. iPSCs cells were detached with TrypLE™ Express Enzyme (ThermoFisher Scientific, Inc.) to obtain a single cell suspension and subsequently labelled for flow cytometry. Cells were fixed and permeabilized using Fix&Perm-Reagent kit(Nordic-MUbio), following the manufacturer’s instructions. Then, blocking solution BSA 5% in PBS was applied for 30 min at RT. Primary antibodies for CD34 (1:50, clone 8G12, Becton, Dickinson and Company), ER (clone SP1-rabbit, ready to use, Ventana, Roche), PR (clone 1E2-rabbit, ready to use, Ventana, Roche) and secondary antibody [1:500; Goat anti-rabbit IgG (H + L) Alexa Fluor-488; ThermoFisher Scientific, Inc.] were added and incubated for 30 min at + 4 °C. Cells were resuspended in PBS and flow cytometry analysis was performed with BD FACSCanto™ II (Becton, Dickinson and Company). Data were collected from at least 1 × 10^4^ cells/sample and elaborated with FlowJo™ v10.8 Software (Tree Star, Inc., Ashland, OR, USA). Data were expressed as signal median fluorescence intensity (ΔMFI) = MFI_stained cells_—MFI_unstained control_.

### Generation of Mammary-like Organoids

Mammary-like organoids were generated following a two-step protocol from iPSCs as previously described by Qu et al. [10]. iPSCs were lifted using TrypLE™ Express Enzyme (ThermoFisher Scientific, Inc.) to obtain a single cell suspension. iPSCs were seeded into AggreWell™ Microwell Plates (StemCell Technologies Inc, CA) following the manufacturer's instructions in order to generate MammoCult-derived embryoid bodies (mEBs) of 2 × 10^3^ cells. The day after generation, mEBs were transferred in ultra-low adherent 6-well plates (Corning Inc, USA) for floating culture in the complete MammoCult medium (StemCell Technologies), supplemented with heparin (4 μg/mL; StemCell Technologies, CA), and hydrocortisone (0.48 μg/mL; StemCell Technologies, CA). 3D culture was performed by embedding 10-days (d) mEBs in mixed Matrigel (2.5 mg/mL; SIAL srl, Italy)/Collagen I (1 mg/ml; Sigma-Aldrich, USA) domes in 6-well-plates (Sarstedt AG & Co. KG, Nümbrecht, Germany). To induce mammary commitment, domes were cultured in complete EpiCult B medium supplemented with parathyroid hormone (pTHrP, 100 ng/ml; Sigma-Aldrich, USA) for 5 days. To induce branch and alveolar differentiation, the domes were cultured in complete EpiCult B medium supplemented with hydrocortisone (1 μg/ml; StemCell Technologies, CA), insulin (10 μg/ml; Sigma-Aldrich, USA), FGF10 (50 ng/ml; Peprotech; ThermoFisher Scientific, Inc.), and HGF (50 ng/ml; Peprotech; ThermoFisher Scientific, Inc.) for the following 20 days.

### Immunohistochemistry (IHC)

mEBs were fixed in 10% formalin for 24 h and were centrifuged at 500 rpm for 5 min. A solution (1:2) of plasma and HemosIL8 RecombiPlasTin 2G (Instrumentation Laboratory, Bedford MA, USA) was added to pellets, mixed until the formation of a clot, and placed into a labelled cassette by paraffin inclusion. The suitability of the paraffin-embedded (FFPE) specimen was evaluated by haematoxylin and eosin (H&E) staining on 2 µm -thick tissue sections. Four micron-thick tissue sections were obtained from formalin-fixed, FFPE blocks. For IHC staining, endogenous peroxidase was blocked by incubation with methanol and hydrogen peroxide 0.03% for 20 min during rehydration. Immunostaining was performed using, CK-PAN (1:200 clone MNF116, Dako, Glostrup, Denmark), CK5 (ready to use, clone D5/16B4, Ventana, Roche, Basel, Switzerland), CK7 (ready to use, clone OV-TL 12/30, Dako, Glostrup, Denmark), CK18 (1:250, clone DC-10, CA, USA), GATA3 (ready to use, clone L50-823, Roche, Basel, Switzerland), TP63 (1:50, clone 4A4 + Y4A3, Cell Marque, Roche, Basel, Switzerland), CD34 (1:50, Leica Biosystems Newcastle Ltd, Newcastle, UK), ER (ready to use, clone SP1-rabbit, ready to use, Ventana, Roche, Basel, Switzerland), PR (ready to use, clone 1E2-rabbit, ready to use, Ventana, Roche, Basel, Switzerland) after pre-treatment with microwave or water bath in or EDTA (ethylenediaminetetraacetic acid) buffer at pH 8. In particular, ER antibody directly binds to ERα isoform, while PR antibody recognizes both isoforms A (PR-A) and B (PR-B) of the progesterone receptor (PR). The reaction was revealed using Novolink Polymer (Leica Microsystems, Wetzlar, Germany) followed by diaminobenzidine (DAB, Dako, Glostrup, Denmark). Finally, the slides were counterstained with Meyer’s Haematoxylin.

### Statistical analysis

Statistical analysis was carried out using GraphPad Prism v8.0 (GraphPad Software, Inc., San Diego, CA, USA) software. One-way ANOVA followed by Tukey’s post-hoc test was used to test the significance among groups. Unpaired to tailed Student’s t-test was used to test the significance between controls. Data were considered statistically significant when p-value ≤ 0.05.

## Results

### Expression of ERα/β and PR mRNA in iPSCs

**Fig. 1:**
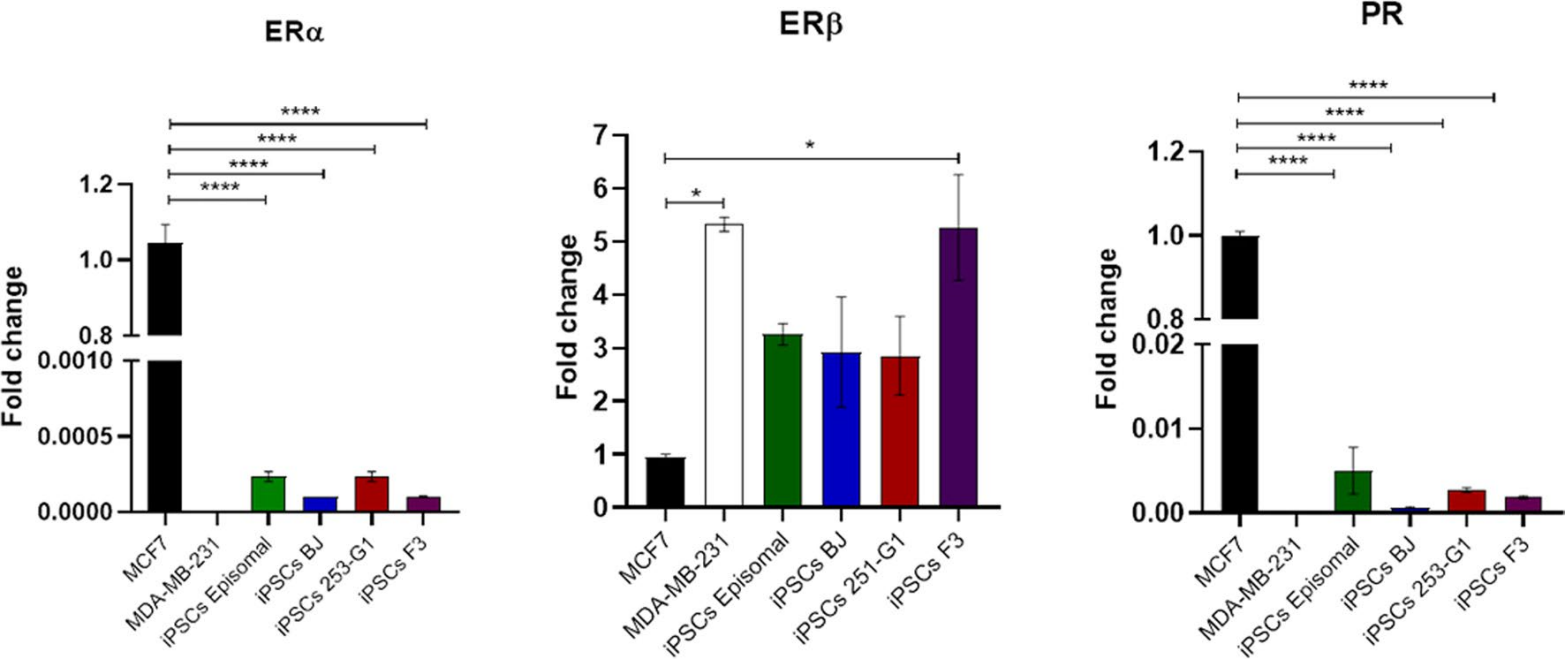
Expression profile of ERα/β and PR receptors in iPSCs compared to MCF7 and MDA-MB-231. **A** ERα mRNA in iPSCs cell lines compared to MCF7 positive and MDA-MB-231 negative control. **B** ERβ mRNA in iPSCs cell lines compared to MCF7 negative and MDA-MB-231 positive control. **C** PR mRNA in iPSCs cell lines compared to MCF7 positive and MDA-MB-231 negative control. Histograms represent fold-change in the gene expression of three independent experiments, while error bars represent ± SEM. One-way ANOVA followed by Tukey’s post-hoc test. **** p < 0.0001

We examined the expression of ERα/β and PR mRNA in four different iPSCs cell lines (Fig. 1) compared to MCF7 (ERα + /ERβ-/PR +) and MDA-MB-231 (ERα-/ERβ + /PR-) human breast cancer cell lines[34]. As shown in Fig. 1A-C, there were no significant differences in the expression of ERα and PR mRNA among the several iPSCs (ERα_Ct mean_ = 34,48 and PR_Ct mean_ = 34,51). Accordingly, the mRNA levels were significantly reduced (*p* < 0.0001) in iPSCs compared to MCF7 positive control cell line (ERα_Ct mean_ = 22,43 and PR_Ct mean_ = 26,33). Interestingly, as shown in Fig. 1B, iPSCs episomal, BJ and 253-G1 showed a trend of upregulation of ERβ (ERβ_Ct mean_ = 32,28) expression at the mRNA level compared to MCF7 (ERβ_Ct mean_ = 35,50). Moreover, iPSCs F3 expressed levels of ERβ mRNA similar to that of MDA-MB-231 (ERβ_Ct mean_ = 31,31), showing a significant upregulation compared to MCF7. These results indicated that iPSCs do not actively transcribe ERα, while express low levels of PR mRNA and active transcription occurs for ERβ.

### Localization of ERα and PR in iPSCs

**Fig. 2:**
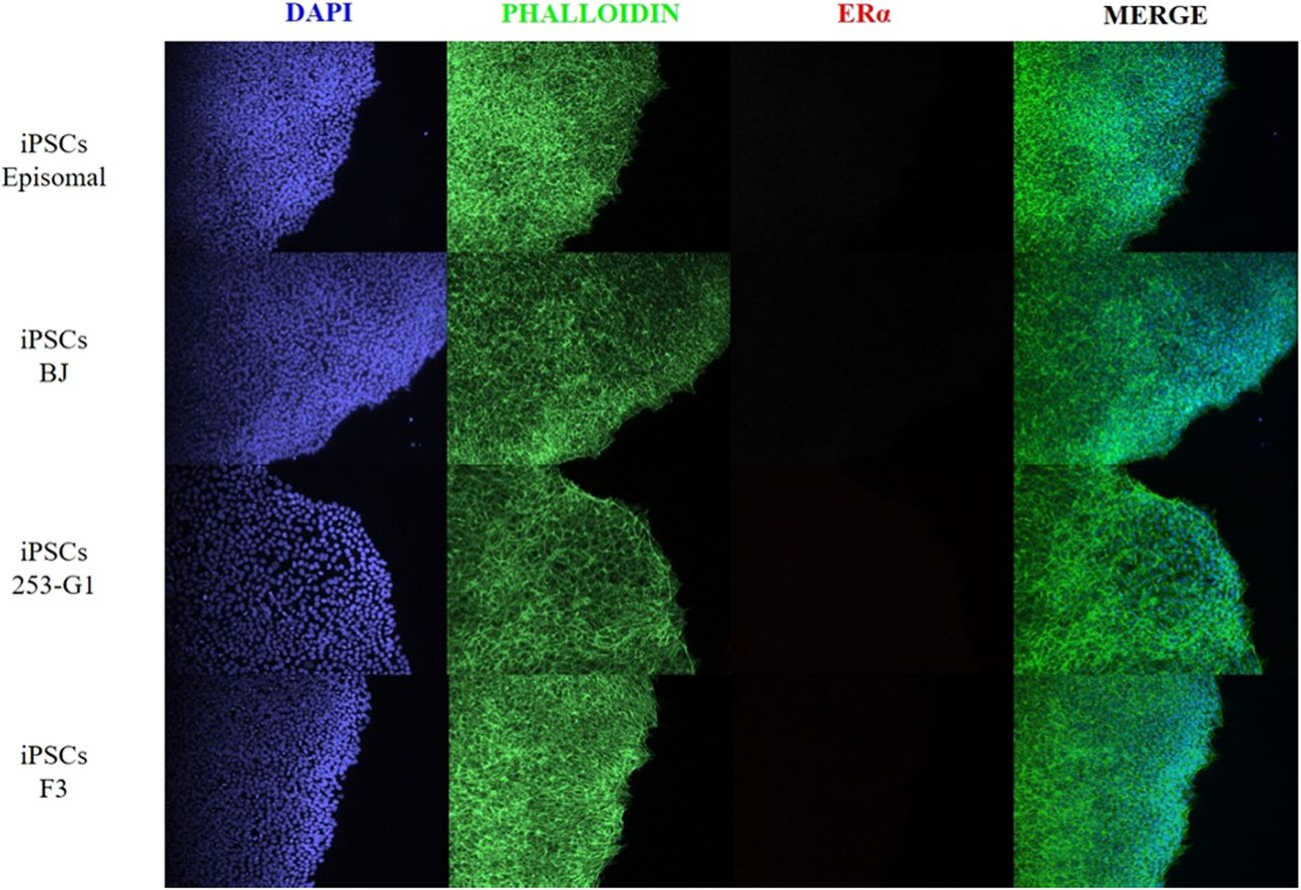
Detection of ERα in iPSCs cell lines. Immunofluorescent (IF) staining for the detection of ERα among the different iPSCs cell lines. Nuclei were counterstained in blue (DAPI), while cytoskeleton in green (phalloidin-488) and ERα in red (Alexa-568). Magnification 10X

**Fig. 3:**
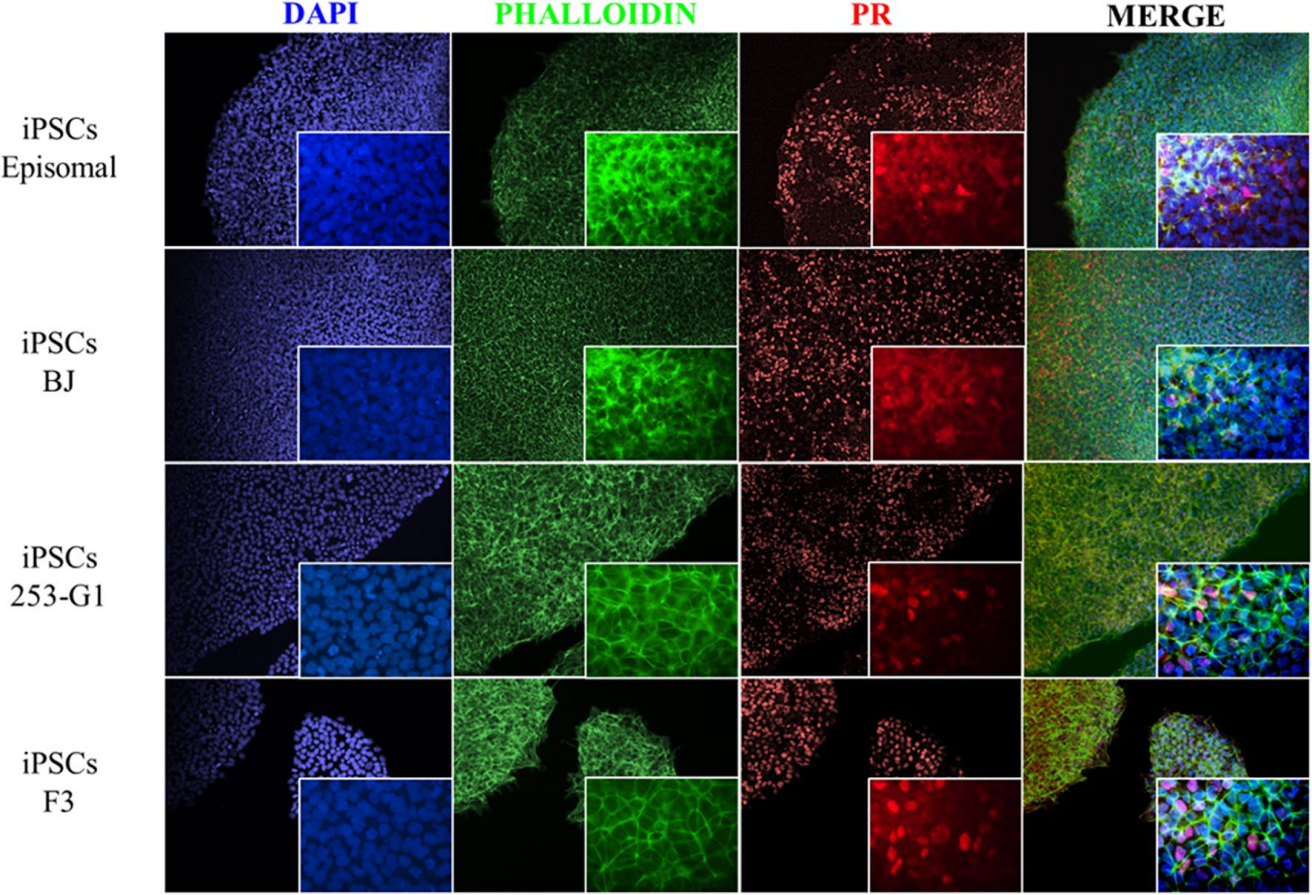
Detection of PR in iPSCs cell lines. Immunofluorescent (IF) staining for the detection of PR among the different iPSCs cell lines. Nuclei were counterstained in blue (DAPI), while cytoskeleton in green (phalloidin-488) and PR in red (Alexa-568). Pictures were acquired at 10X (background) and 60X (foreground) magnification respectively. Representative images of at least 4 independent fields of two independent experiments

As mRNA levels not always predict protein expression levels, we further performed IF analysis (Figs. 2 and 3) on the different iPSCs cells. We used specific antibodies routinely used in the diagnostic microscopy practice (Suppl. Fig. 1). In particular, as reported in Materials&Methods, ER antibody directly binds to ERα isoform, while PR antibody recognizes both isoforms A (PR-A) and B (PR-B) of the progesterone receptor (PR). As shown in Fig. 2, ERα protein was not detectable in iPSCs colonies.

Surprisingly, as shown in Fig. 3, we detected the expression of PR protein in the nucleus of all the iPSCs cell lines generated from different parental cells with several reprogramming methods (Table 1). These results suggested that active translation occurred.

### Expression of ERα/β and PR in parental cells

**Fig. 4:**
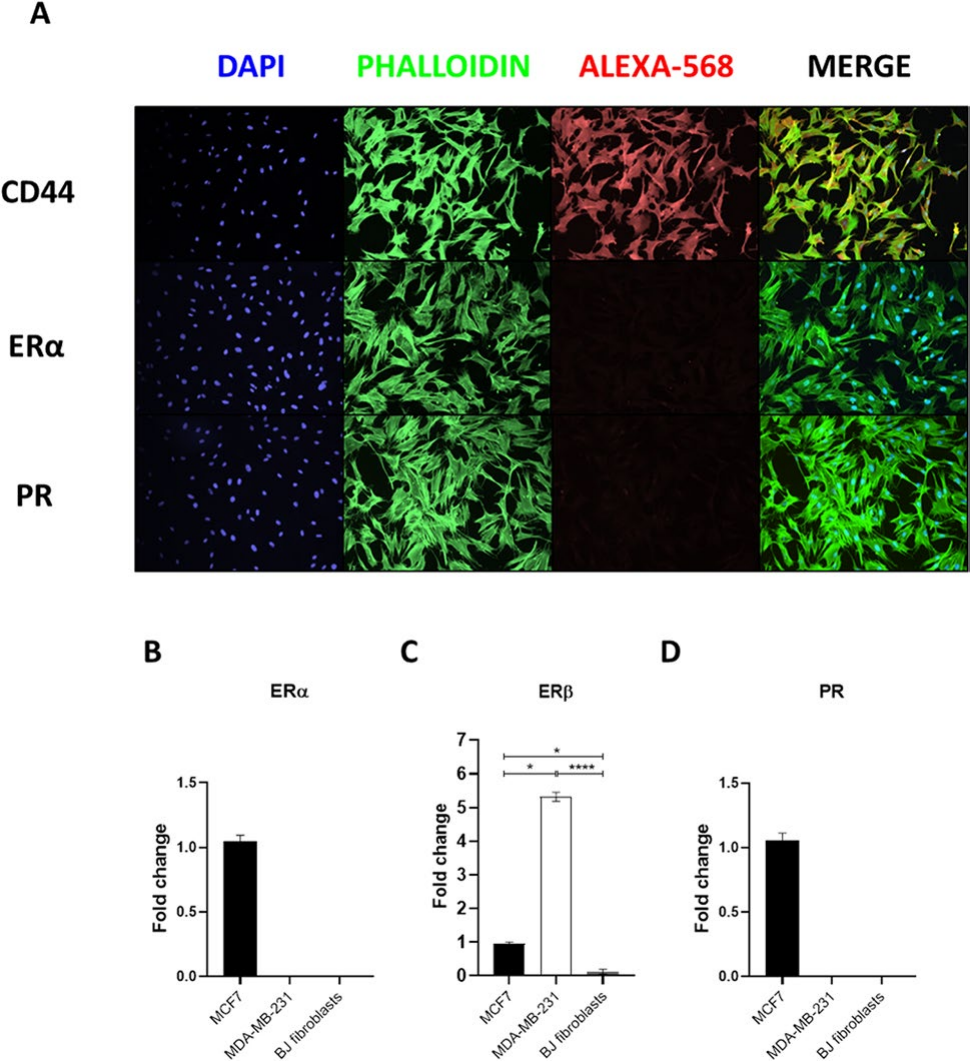
CD44, ERα/β and PR in BJ human foreskin fibroblasts. **A** Immunofluorescent (IF) staining. Nuclei were counterstained in blue (DAPI) and cytoskeleton in green (phalloidin-488), while CD44, ERα and PR in red (Alexa-568). Magnification 10X. **B**-**C** Gene expression analysis of ERα/β in MCF7, MDA-MB-231 and BJ fibroblasts. **D** Gene expression analysis of PR in MCF7, MDA-MB-231 and BJ fibroblasts. Histograms represent fold-change in the gene expression of three independent experiments, while error bars represent ± SEM

In order to determine the moment in which the PR protein expression arose in iPSCs, we extended the analysis of the expression pattern to the precursor cells. In particular, as three out of four iPSCs cell lines where generated from fibroblasts (Table 1), we performed IF analysis of ERα and PR proteins on foreskin BJ parental fibroblasts. As shown in Fig. 4A, BJ fibroblasts, expressing CD44 cell surface adhesion glycoprotein marker, lack the expression of ERα and PR proteins. These results were also confirmed by gene expression analysis. As show in Fig. 4B-D, ERα and PR were not expressed in BJ fibroblasts compared to MCF7 positive and MDA-MB-231 negative controls respectively, while ERβ gene expression was significantly downregulated (Fig. 4C) compared to MCF7 (*p* < 0.05*) and MDA-MB-231 (*p* < 0.0001****).

**Fig. 5:**
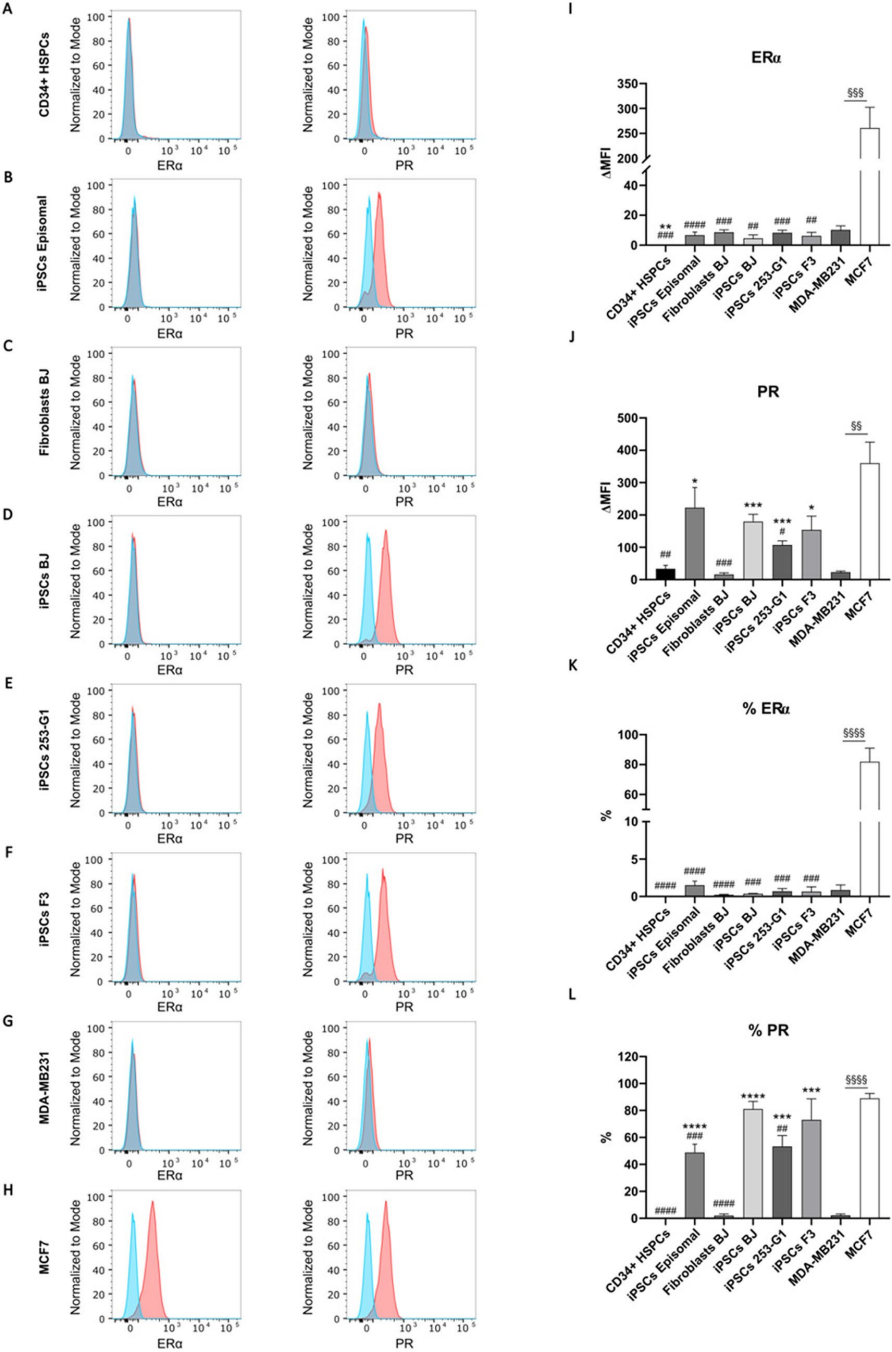
Flow cytometry analysis for the detection of ERα and PR in mobilized-PB, fibroblasts, and iPSCs. **A**-**H**: representative images of ERα (left) and PR (right) expression in G-CSF mobilized-PB (gated on CD34 + HSPCs, **A**, Episomal iPSCs (**B**), BJ fibroblasts (**C**), BJ iPSCs (**D**), 253-G1 iPSCs (**E**), F3 iPSCs (**F**), and in MDA-MB231 negative control (**G**) and MCF7 positive control (**H**); red = aspecific fluorescence, blue = FITC-labeled target. I-L: Histograms representing either ΔMFI = median fluorescence intensity (**I**,**J**) or percentage (**K**,**L**) of ERα (**I**,**K**) and PR expression (**J**,**L**) in G-CSF mobilized-PB (gated on CD34 + HSPCs), Episomal iPSCs, BJ fibroblasts, BJ iPSCs, 253-G1 iPSCs, F3 iPSCs, and in MDA-MB231 negative control and MCF7 positive control. Bars represent the mean ± SEM from at least three independent experiments.*, # *p* < 0.05, **, ##, §§ *p* < 0.01, ***, ###, §§§ *p* < 0.001, ****, ####, §§§§ *p* < 0.0001; * vs MDA-MB231; # vs MCF7; § MDA-MB231 vs MCF7

Furthermore, as previously reported (Table 1; Suppl. Fig. 2), iPSCs episomal cell line was generated with a viral-integration-free method from cord blood-derived CD34 + progenitor cells. Blood cells are the most used cell types for reprogramming. In order to explore whether HSPCs would express ERα and PR, we further performed flow cytometry analysis on a G-CSF mobilized-PB control group. As shown in Fig. 5A (left panel), ERα was not detectable in CD34 + HSPCs. Lack of expression of ERα in episomal iPSCs as well as in all iPSCs and BJ fibroblasts was also confirmed (Fig. 5B-H, left panel) compared to MCF7 positive and MDA-MB-231 negative controls (Fig. 5I-K). Strikingly, we did not detect the expression of PR (Fig. 5A, right panel) in CD34 + HSPCs cells (0.18%) and BJ fibroblasts (2.08%), while we observed that an average of ~ 65% of iPSCs expressed PR protein (Fig. 5J-L). Interestingly, a direct observation of PR upregulation came from iPSCs generated from BJ fibroblasts (Figs. 4 and 5D-J-L).

Taken together these results suggested that PR protein is not detectable in HSPCs and fibroblasts, while its expression arose once somatic cells are reprogrammed to iPSCs.

## Longitudinal modulation of PR receptor during iPSCs mammary-like organoids generation

**Fig. 6:**
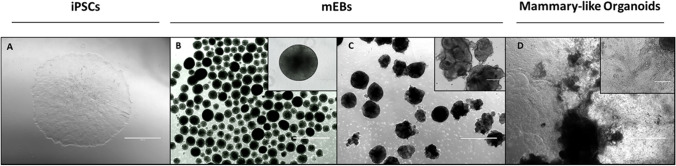
Representative images of morphological changes during mammary-like organoids generation from iPSCs. **A** iPSCs colony; **B** 1-d mEBs differentiation; **C** 10-d mEBs differentiation; **D** Mammary-like organoids. Pictures were acquired at 4X (background) and 20X (foreground) magnification respectively

To understand whether PR expression may have functional implications during iPSCs differentiation, we generated mammary-like organoids from iPSCs Episomal and BJ, as representative CD34 + progenitors and fibroblasts derived-iPSCs, respectively. As shown in Fig. 6, iPSCs (Fig. 6A) were addressed to form mEBs (Fig. 6B). Branching-morphogenesis and alveolar mammary-like structures developed from 10-days mEBs embedded in mixed gel (Fig. 6C), and the morphology got more pronunced during the following 20-days of differentiation (Fig. 6D).

**Fig. 7:**
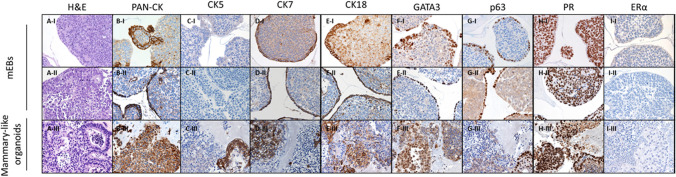
H&E and IHC staining. A) H&E stain; B) PAN-CK; C-D-F) Luminal (CK5/7 + and GATA3 +) and E–G) basal cells markers (CK18 + and TP63 +); H) PR and I) ERα. Representative images of mEBs obtained from iPSCs Episomal (I); mEBs obtained from iPSCs BJ (II); mammary-like organoids (III) differentiation

As shown in Fig. 7, IHC showed positive staining for luminal (CK5/7 + and GATA3 +) and basal markers (CK18 + and TP63 +), collectively highlighting that mammary-like organoids were generated. In particular, PR staining for mammary-like organoids showed positive protein expression. We did not detect ERα protein expression as cells in active proliferation down-modulate expression of ERα during mammary gland development [35–40].

**Fig. 8:**
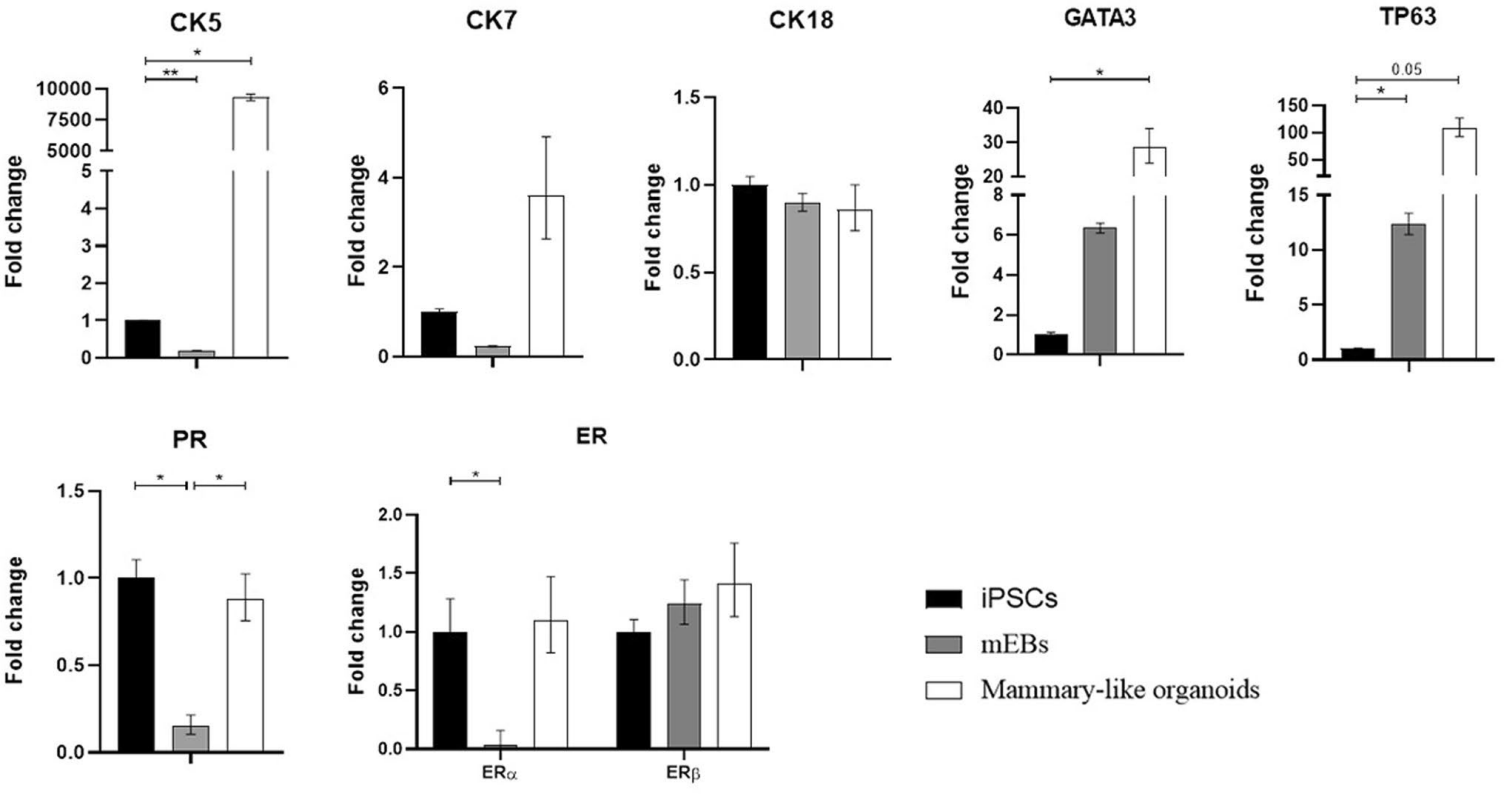
Gene expression analysis of luminal (*CK5/7* + and *GATA3* +) and basal markers (*CK18* + and *TP63* +) markers, *PR, ERα* and *ERβ* in iPSCs, 10d-mEBs and 20-d mammary-like organoids. Histograms represent fold-change in the gene expression, while error bars represent ± SEM. *p < 0.05, **p < 0.01

These results were also supported by gene expression analysis. The expression of stemness-pluripotency marker genes (*NANOG, OCT4*) was significantly reduced (Suppl. Fig. 3). On the other hand, as shown in Fig. 8, the expression of luminal (*CK5/7* and *GATA3*) and basal markers (*TP63)* was upregulated during iPSCs differentiation to mammary-like organoids generation. *CK18* expression was unchanged. These results indicated the co-existance of several cellular phenotypes (luminal and basal cells).

In particular, we observed that the gene expression of *PR* was significantly reduced in the early stage of differentiation from iPSCs to 10-d mEBs. Accordingly, a similar reduction was observed in *ERα* expression; *ERβ* was not affected, on the contrary it showed a trend of upregulation during mEBs maturation into mammary-like organoids. Moreover, considering PR downstream target genes, *CK5* in the early stage was significantly low expressed in iPSCs and 10-d mEBs compared to mammary-like organoids, where its expression was remakably upregulated during the late stage of differentiation. Regarding *GATA3,* undifferentiated iPSCs expressing elevated levels of *PR*, showed a low expression of *GATA3*. On the other hand, during the early stage of 10d-mEBs differentiation, *PR* and *GATA3* showed opposite trend: the expression of *PR* was reduced while the expression of *GATA3* showed a trend of upregulation, reaching a significant expression during mammary-like organoids differentiation in late stage of 20-d maturation. Subsequently, *PR* expression was restored in 20-d mammary-like organoids to levels similar to that of iPSCs. The concomitant expression of *PR* and *GATA3* in 20-d mammary-like organoids could be explained due to their functional role during development similar to that of the human mammary gland.

## Discussion

Ex vivo culture of embryonic pluripotent stem cell (ESCs) that can produce all cell types in the adult body was established 40 years ago and has provided an important understanding of developmental biology [41]. Reprogramming technologies enable cells to enter an ESCs-like state, resulting in the generation of iPSCs. Studies so far highlighted that reprogramming is a complex process characterized by unique gene expression patterns dealing with chromatin remodeling and epigenetic modifications, proliferation and cellular senescence resulting in complex morphological and functional changes meant to a specific cellular phenotype [42–48]. Several analysis indicated that iPSCs share many key properties with ESCs as pluripotency, self-renewal, EBs formation and similar gene expression profile [49].

Steroid hormones, as estrogen and progesterone, play different roles in particular during embryonic development. Accordingly, steroids hormone-related proteins as estrogen receptors (ERα/β) and progesterone receptor (PR) were reported to be expressed in ESCs [50] and during early development in mice through the blastocyst stage [51]. In particular, ERβ importance in self-renewal and pluripotency has been further elucidated[52, 53]. Progesterone has been reported to be essential for the differentiation of ESCs during human embryonic development [54], the action of which is mediated by PR-A expressed in ESCs [55–57]. In addition progesterone also induces [58] or inhibits [59] the differentiation of ESCs into specific lineages, as well as the development and physiology of steroid-hormones responsive organs [60–64].

PR consists of two main isoforms, PR-A and PR-B and their transcription is controlled by distinct estrogen-inducible promoters with alternative AUG initiation codons; hence PRs are thought to be direct targets of ERs [65]. A functional difference between PR-A and PR-B is that PR-A can act as a dominant repressor of both PR-B and ER in a promoter and cell-type specific manner [66, 67]. Interestingly, the DNA-repair tumor suppressor protein BRCA1 (*BReast CAncer gene 1*) has been shown to interact with and to regulate ERα and PR transcriptional activation [68–70].

In this context, the comprehension of the molecular events leading to iPSCs reprogramming would improve the development of iPSCs-based disease cellular models in particular for those related to steroid hormone cellular response such as reproductive organs (i.e. ovaries, breast). Cellular reprogramming is a complex event involving the activation and repression of several specific genes and therefore the regulation of the related proteins. The identity of the cell of origin that undergoes reprogramming into an iPSC as well as the technology performed are also important for iPSCs-based applications. Episomal-vectors are a non-integrating reprogramming system introduced into the cell by electroporation [71]. The vectors replicate only once per cell cycle, with activation of replication by binding of multiple EBNA-1 homodimers to oriP within the nucleus [72]. The Episomal iPSC reprogramming vectors are a well-described system for producing transgene-free, virus-free, iPSCs from a number of different somatic cell types [72]. Sendai-virus is a single stranded, negative sense RNA virus (ssRNA-), member of the *Paramyxoviridae* family of viruses, which vertebrates serve as natural hosts. Sendai replicates in the cytoplasm independently of the cell cycle and transduces a wide range of somatic cell types [73]. Retrovirus and lentivirus are DNA host-integrated vectors, prone to incomplete silencing of reprogramming transgenes, which leads to incomplete reprogramming. Additionally, lingering expression or re-expression of viral transgenes as well as insertional mutagenesis and random integration could interfere with iPSC-derived cells differentiation potential [74].

Blood cells and skin fibroblasts are commonly used cell types for reprogramming. Although assumed to be solely a hematopoietic stem cell (HSCs) marker, the detection of CD34 in BM or PB samples represents a hematopoietic stem/progenitor mix, of which the majority of cells are progenitors. Indeed, CD34 is a single-pass transmembrane sialomucin protein [75–81], widely used as a marker of HSCs [82–84], vascular endothelial cells [85, 86] and progenitor cells (progenitors for mast cells (pMC) and eosinophils (pEo), in particular, can exit the BM as CD34 + precursors) [87]. In the BM, the early endothelial progenitor cells (EPC) are also characterized by the expression of CD34, CD133 and the VEGFR-2 [88]. In the PB of adults, more mature EPC are found that have lost CD133, but are still positive for CD34 and VEGFR-2 [89]. Human HSCs could be further separated from CD34 + progenitor cells by low expression of CD90 and a lack of expression of CD38, human leukocyte antigen-DR (HLA-DR), and a panel of mature hematopoietic lineage markers (Lin −) [90]. HSPCs may maintain greater genomic stability than terminally differentiated somatic cells [12], moreover they lack V(D)J rearrangements of committed T and B cells [11, 13], representing a suitable cell to be reprogrammed. Contrary from skin fibroblast, easy to obtain by skin biopsy, CD34 + HSPCs, despite being highly proliferative and ready for efficient reprogramming after 2–5 days culture, are rare in adult PB (< 0.01%), unless the donors have been treated with a stem cell mobilization regimen as G-CSF [91, 92].

Nakada et al. [93] reported that although males and females mice have similar basal numbers of HSCs and their multipotent progenitor cells (MPPs), females exhibited increased frequency of proliferation of these cells without depletion of the stem cell pool. This indicated that female HSCs underwent more frequent self-renewing divisions. The enhanced proliferation of HSCs in females’ mice was driven by endogenous estrogens and mediated mainly by intrinsic ERα, which was highly expressed in HSCs. During pregnancy, more HSCs were detected in the BM and spleen relative to non-pregnant female mice. Significant increases in spleen cellularity, erythropoiesis, and myelopoiesis were also observed during pregnancy with elevated estrogen levels, highlighting the importance of sex hormones in HSCs activity to respond to increased oxygen consumption and produce more erythrocytes. Nakada et al. detected little or no ER, PR or androgen receptor expression in HSCs (CD150 + CD48-Lin-Sca-1 + c-kit +) and MPPs [93]. However, in a murine BM–derived HSPCs subset (Sca-1 + Lin-CD45 +) [56] and a CD34 + Lin-CD45 + population isolated from human umbilical cord blood [57], expressions of receptors for estrogens, androgen, and PR, as well as FSH, LH, and prolactin, were detected [56, 57].

In the present study, we did not observe the expression of ERα and PR in CD34 + HSPCs, as well as in skin fibroblasts. We did not detect a consistent amount of ERα and PR mRNAs. On the contrary, ERβ mRNA was upregulated in the different iPSCs. Accordingly, ERβ has been reported to be also required and sufficient to activate formative genes[94]. Besides localization, the PR upregulation was strongly supported also by flow cytometry analysis. Moreover, we showed PR expression is dynamic. Indeed, longitudinal expression of PR expression was also consistent with the concomitant regulation of PR-downstream effector genes *CK5*[95] and *GATA3*[96] during iPSCs-mammary-like organoids development. *GATA3* expression is critical for the luminal differentiation of mammary epithelial cells and in the morphogenesis of the mammary gland. Indeed, PR activation downregulates *GATA3* by transcriptional repression [96–98]. The absence of expression of ER and PR in HSPCs and skin fibroblasts could be explained as they are differentiated cells, while a subpopulation of stem cells amongst them migrating primordial germ cells expand as iPCSs cells[99, 100]. PR expression, therefore, might arise in the early stages during reprogramming into iPSCs, where cells acquire features similar to that of ESCs. Collectively these data would indicate that independently from the cell of origin (CD34 + or skin fibroblasts) to be reprogrammed as well as the technology used (episomal-vectors, Sendai virus, retrovirus, or lentivirus transduction) PR may play a role during cell reprogramming into iPSCs. Furthermore, as PR is already expressed in iPSCs, it could not be used as a specific marker of iPSCs-cell based differentiation. Indeed, these observations are useful for wider considerations in iPSCs-based disease models, especially for those involving PR-responsive organs (i.e. mammary-like/ovaries). Nevertheless, PR expression is regulated during iPSCs differentiation. Finally, this study could be a starting point to better comprehend the molecular mechanisms involved in cell development and cellular response to treatments.

## Conclusions

In conclusion, with the present study we demonstrated for the first time the presence of progesterone receptor after reprogramming in iPSCs, underling their close relation to ESCs, and opening a new scenario on iPSCs and their applications. Further studies will be addressed to determine the proper resolution of PR-isoform (PR-A or –B), as well as the functional role of PR in iPSCs cells and the signaling pathways involved.

## Supplementary Information

Below is the link to the electronic supplementary material.

ESM 1 (PDF 484 KB)

## Declarations

### Data Availability

The data supporting the findings of this study are contained within the contents of this article. The datasets generated during this study will be freely provided by the corresponding author upon request.
