# Supplementary material for: Progesterone receptor is constitutively expressed in induced Pluripotent Stem Cells (iPSCs)
Source: Stem Cell Rev Rep. 2024 Aug 22;20(8):2303–17. doi: 10.1007/s12015-024-10776-6 (PMC11554879; doi:10.1007/s12015-024-10776-6)
Supplement: Supplementary file 1 — (PDF 484 KB) [file 12015_2024_10776_MOESM1_ESM.pdf]

Supplementary Figure 1

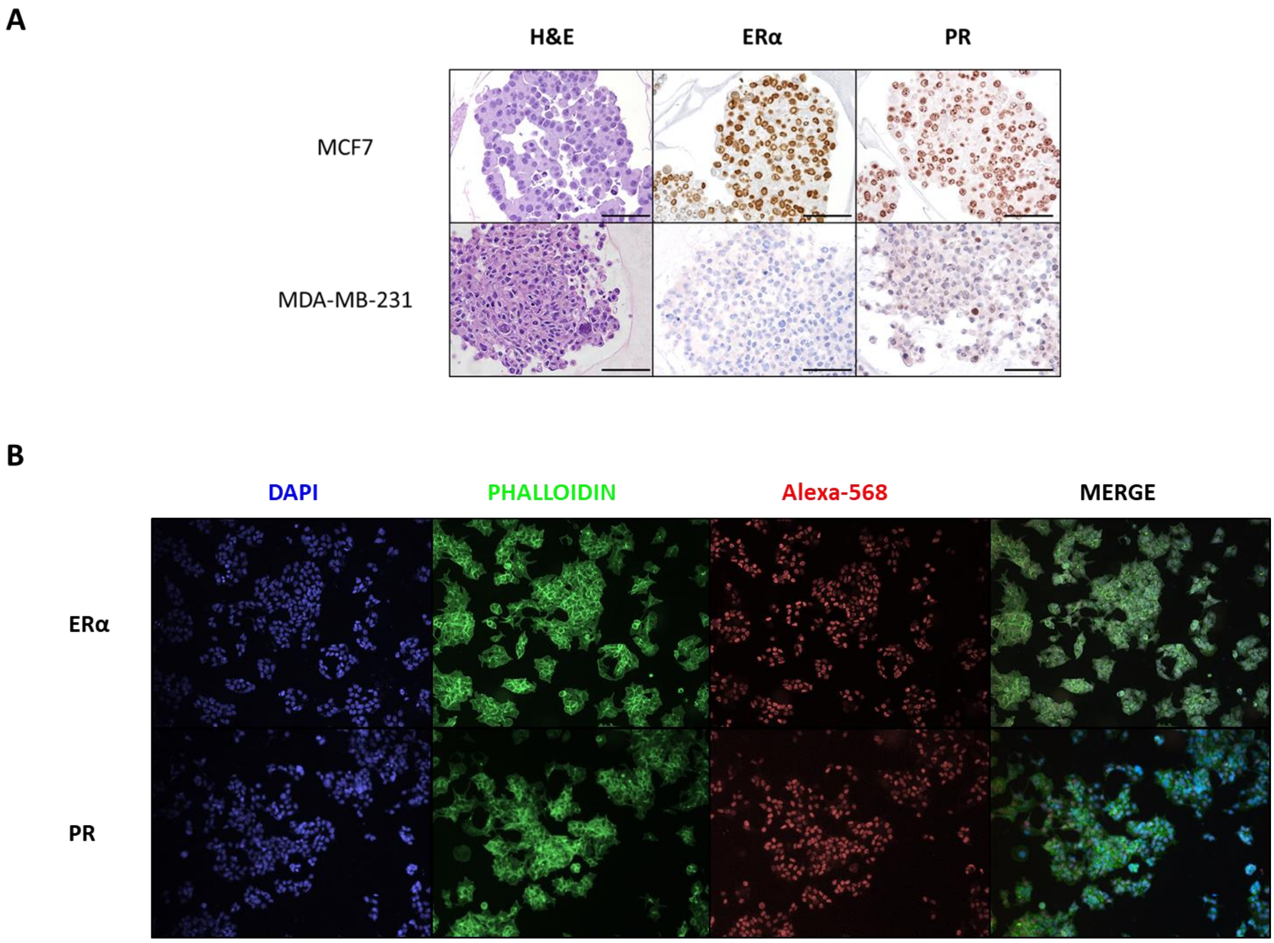

Supplementary Figure 1. A) H&E and IHC staining for the detection of ERα and PR in mammospheres. Mammospheres were generated from MCF7 (ER+, PR+) and MDA-MB-231 (ER-, PR-) cells in complete MammoCult medium (STEMCELL Technologies Inc., Vancouver, B.C.). B) IF staining for ER and PR in MCF7 breast cancer cell line positive control. Antibodies used for the detection of ERα and PR were listed in Materials and Methods section. Scale bar is 100µm.

Supplementary Figure 2

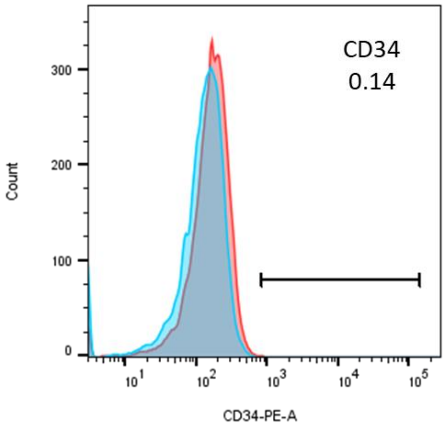

Supplementary Figure 2. Detection of CD34 in iPSCs. Flow cytometry analysis for CD34 in iPSCs episomal cell line. Red indicated aspecific fluorescence, while blue indicated labelled-PE target.

Supplementary Figure 3

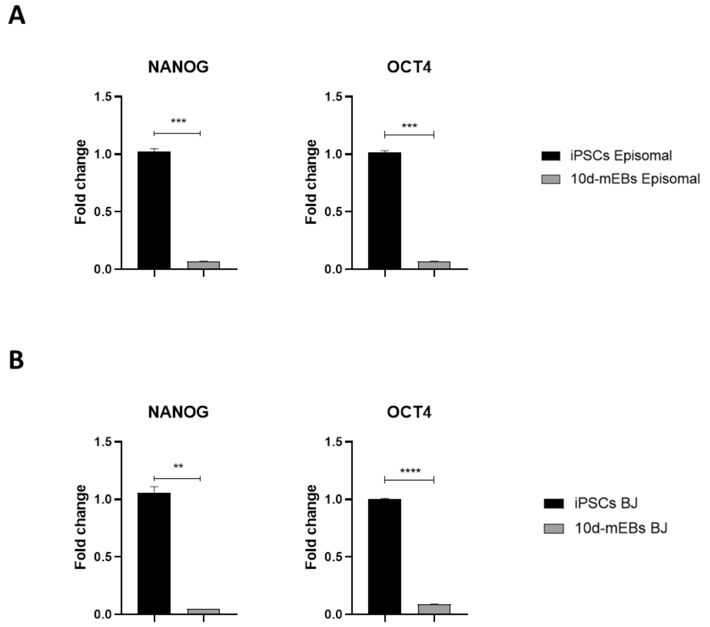

Supplementary Figure 3. Expression of Stemness marker genes NANOG, OCT4. A) Episomal B) BJ. Histograms represent fold-change in the gene expression, while error bars represent ± SEM. Unpaired to tailed Student's t-test. **$p<0.01$, ***$p<0.001$, ****$p<0.0001$.
